# Use of dalbavancin in infective endocarditis: a case series

**DOI:** 10.1093/jacamr/dlab099

**Published:** 2021-08-12

**Authors:** Achyut Guleri, Ranjit More, Rashmi Sharma, Michelle Wong, Amr Abdelrahman

**Affiliations:** 1 Lancashire Cardiac Centre, Blackpool Teaching Hospitals NHS Foundation Trust, Blackpool, UK; 2 Blackpool Teaching Hospitals NHS Foundation Trust, Blackpool, UK

## Abstract

**Background:**

Infective endocarditis, typically caused by Gram-positive organisms such as viridans group streptococci and *Staphylococcus aureus*, is associated with high mortality and morbidity and requires aggressive, prolonged antimicrobial treatment and sometimes surgery. Dalbavancin, a lipoglycopeptide active against Gram-positive pathogens, has a long half-life, which allows IV treatment as one dose or two doses with a prolonged interval, offering personalized treatment for complex psychosocial situations or facilitating early discharge. In the absence of randomized controlled trials in infective endocarditis, current evidence derives from real-world case series involving off-licence use. The Austrian Society for Infectious Disease and Tropical Medicine includes dalbavancin as an option for infective endocarditis.

**Objectives:**

This retrospective case series reports use of dalbavancin in a small cohort of patients with infective endocarditis treated at Lancashire Cardiac Centre, Blackpool Teaching Hospitals Foundation Trust, UK.

**Results:**

The pharmacy database included 11 patients in whom dalbavancin was used to address either complex psychosocial circumstances or the need for early discharge. The endocarditis multidisciplinary team selected dalbavancin from available treatment options. Structures affected by infective endocarditis included aortic, mitral and tricuspid valves; aortic composite grafts; implantable cardioverter defibrillator leads; and prosthetic aortic valves. Eight patients underwent surgery; three were managed conservatively with antibiotics. Dalbavancin was curative in all but one patient, whose signs and symptoms of infection improved. No patients developed adverse reactions.

**Conclusions:**

Dalbavancin is an alternative treatment option at hospital discharge when conventional antibiotics may not be suitable due to complex psychosocial issues or early discharge is required.

## Introduction

Infective endocarditis, predominantly caused by Gram-positive pathogens such as viridans group streptococci and *Staphylococcus aureus*,^1^^,^^2^ is associated with in-hospital mortality of 10%–30% and non-fatal complications such as acute stroke.^1^^,^^3^^,^^4^ Management may require aggressive and prolonged antimicrobial treatment and/or surgery.^4^ Conventional antibiotics with relatively short half-lives may not be suitable for patients requiring early discharge or those with complex psychosocial situations such as IV drug use, non-compliance or personal/social issues, so alternatives are needed.

Dalbavancin is a lipoglycopeptide antibiotic approved in the USA and Europe for Gram-positive acute bacterial skin and skin structure infections in adults.^4–7^ It has *in vitro* activity against various Gram-positive pathogens involved in infective endocarditis,^8^ but randomized controlled trials excluded patients with infective endocarditis and infected devices.^9^^,^^10^ However, dalbavancin was effective in animal models of infective endocarditis and *in vitro* assays against Gram-positive organisms, including MRSA, viridans-group streptococci, *Enterococcus faecalis* and *Enterococcus faecium*.^11–14^ Most clinical evidence for infective endocarditis derives from retrospective case series involving off-licence use, with efficacy of 81.4%–96.7% against a variety of Gram-positive organisms.^15–17^ European and UK guidelines for dalbavancin in infective endocarditis are pending review, but the Austrian Society for Infectious Disease and Tropical Medicine includes dalbavancin as an option for infective endocarditis in outpatient parenteral antibiotic therapy (OPAT) settings.^18^^,^^19^

Our case series adds to the evidence by reporting use of dalbavancin in a small cohort of patients with infective endocarditis at a single centre in the UK.

## Methods

The hospital pharmacy database at Lancashire Cardiac Centre, Blackpool Teaching Hospitals Foundation Trust, UK, was reviewed to retrospectively identify all patients treated with dalbavancin for infective endocarditis between 2017 and 2019. We extracted demographics; previous medical history; risk factors; laboratory findings; previous antibiotics; pathogens isolated through blood culture and 16S ribosomal PCR; and use of and outcomes from dalbavancin (Table 1).

**Table 1.** Patient histories

Characteristic	Patient										
	1	2	3	4	5	6	7	8	9	10	11
Age at treatment, years	41	64	59	79	31	64	73	80	72	81	79
Sex	male	male	male	female	male	male	male	male	male	male	male
Previous cardiac surgery	no	no	no	no	no	AVR	no	AVR	AVR	no	no
Comorbidities, Charlson	CCF, renal disease, hypertension	none	hypertension, CCF, depression	MI, CCF, renal disease, hypertension, cancer	severe liver disease	CCF, PVD, renal disease, anticoagulation	none	MI, hypertension, stroke	MI, renal disease, CCF, hypertension, diabetes	renal disease, depression, cancer	mild liver disease, hypertension, cancer
Risk factors for IE	IVDU	degenerative MV	none	ICD implant	IVDU	AVR, aortic composite graft	none	AVR	AVR	none	none
CIED/valve(s) involved in IE	MV, AV	MV	AV	ICD lead	TV	aortic composite graft	AV	AVR	AVR	aortic	aortic
Hb on admission, g/L	77	129	82	100	81	113	102	88	114	110	106
Albumin, g/L	28	38	23	39	19	40	39	24	32	23	29
AST, U/L	38	26	485	33	49	16	25	43	46	11	16
WCC, 10^9^ cells/L	11	5	14	15	12	6	1.2	20	13	7	6
CRP, ng/mL											
admission	61	150	174	118	176	12	188	135	253	212	88
discharge/ 6 weeks after admission	26	15	16	3	8	4	15	75	4	8	8
Surgery for IE	MVR, AVR, TV repair	MV repair 1 month after discharge	AVR, aorto- atrio fistula repair	ICD extraction	none	none	AVR	new AVR, aorto-atrio fistula repair	none	AVR	AVR
IE complications	acute renal failure	none	aorto-atrio fistula	discitis	cavitating lung lesions	none	none	aorto-atrio fistula	none	none	none
Pathogen growth											
blood cultures	nil	*S. gallolyticus*	*S. mitis*	*E. faecalis*	MSSA	MSSA	*S. oralis*	*E. faecalis*	*S. oralis*	*E. faecalis*	*E. faecalis*
PCR	MSSA	not operated, no sample	*S. mitis*	not operated, no sample	not operated, no sample	not operated, no sample	*S. oralis*	*E. faecalis*	not operated, no sample	*E. faecalis*	*E. faecalis*
Antibiotics given prior to DAL for IE	VAN, GEN, CXM, RIF	benzylpenicillin initially, then AMX	VAN, GEN, discharged on AMX	AMX, CRO	flucloxacillin, RIF	flucloxacillin, RIF, CEC	AMX, LZD	AMX, CRO, LZD	VAN, AMX, GEN	AMC, AMX	AMX, CRO
DAL dose given	1.5 g IV ×2, 1 week apart	1.5 g IV pre- discharge	1.5 g IV	1.5 g IV	1.5 g IV ×2, 1 week apart	1.5 g IV × 2, 1 week apart	1.5 g IV	1.5 g IV ×2, 1 week apart	1.5 g IV × 2, 1 week apart	1.5 g IV	1.5 g IV ×2, 1 week apart
Reason for DAL	to allow early discharge in IVDU patient threatening to self-discharge	early discharge	early discharge	early discharge	IVDU insisting on early discharge	early discharge	early discharge	early discharge/ prosthetic valve infection in elderly patient	early discharge	early discharge	unable to attend OPAT
Adverse reaction to DAL	no	no	no	no	no	no	no	no	no	no	no
Outcome	cure	cure	cure	cure	cure	cure	cure	cure	cure	cure	symptom improvement
12-month follow-up	well	well	well	well	uncontactable but alive	well	well	well	well	well	died 10 months after surgery of advanced bladder cancer

‘Not operated, no sample’ indicates conservative management/surgically very high risk to operate. ‘Cure’ indicates clinical and microbiological resolution.

AMC, co-amoxiclav; AMX, amoxicillin; AV, aortic valve; AVR, aortic valve replacement; CCF, congestive cardiac failure; CEC, cefaclor; CIED, cardiovascular implantable electronic device; CRO, ceftriaxone; CRP, C-reactive protein; CXM, cefuroxime; DAL, dalbavancin; GEN, gentamicin; Hb, haemoglobin; ICD, implantable cardioverter defibrillator; IE, infective endocarditis; LZD, linezolid; MI, myocardial infarction; MV, mitral valve; MVR, mitral valve replacement; OPAT, outpatient parenteral antibiotic therapy; PVD, peripheral vascular disease; RIF, rifampicin; TV, tricuspid valve; VAN, vancomycin; WCC, white cell count.

### Ethics

As this was a retrospective case series, our R&D department confirmed National Research Ethical approval is not required for this service evaluation.

## Results and discussion

### Population

A total of 11 patients treated with dalbavancin for infective endocarditis between 2017 and 2019 were identified. The multidisciplinary team selected dalbavancin with or without oral antibiotics at discharge because its long half-life could facilitate early hospital discharge or address complex psychosocial circumstances impeding treatment. The manufacturer of dalbavancin provided guidance on dosing. As treatment was off licence, patient consent was obtained and documented. Inpatient antibiotics were not continued with dalbavancin. Oral antibiotics were used in combination, as required and clinically indicated based on microbiological results. Blood tests, echocardiograms and monitoring at end of treatment and follow-up were per standard of care.

### Case histories

#### Patient 1

A 41-year-old male IVDU with infective endocarditis of the aortic and mitral valves was transferred to our centre from the parent hospital and underwent aortic and mitral valve replacement and tricuspid valve annuloplasty. Past medical history included admission with sepsis, MSSA-positive blood culture and partial treatment due to self-discharge against medical advice. He had no prior history of infective endocarditis or cardiac surgery. Valvular tissue was positive for MSSA by 16S ribosomal DNA. Preoperatively, he received vancomycin, gentamicin, cefuroxime and rifampicin over 2 weeks. Post-operatively, he indicated his intention to self-discharge. The multidisciplinary team decided on two doses of dalbavancin 1.5 g IV 1 week apart plus rifampicin 600 mg orally twice daily for 4 weeks as an effective combination to complete treatment for his deep-seated infection. Follow-up showed normal inflammatory markers, negative blood culture and normal echocardiograms to 12 months, when the patient was well and cured (defined as microbiological and clinical resolution).

#### Patient 2

A 64-year-old male presented with *Streptococcus gallolyticus*-associated mitral valve endocarditis. He had no previous cardiac surgery or comorbidities. He was treated with benzylpenicillin as an inpatient. Once medically stable, he was discharged after receiving a single dose of 1.5 g dalbavancin and oral amoxicillin to complete 6 weeks of total treatment. He underwent elective mitral valve repair on completion of treatment.

#### Patient 3

A 59-year-old male with a history of hypertension, congestive cardiac failure and depression presented with *Streptococcus mitis*-associated aortic valve endocarditis. He underwent aortic valve replacement and repair of an aorto-atrio fistula complicating the infection. He was initially treated with vancomycin and gentamicin. Once medically stable, he was discharged after single dose of 1.5 g dalbavancin and oral amoxicillin to complete 6 weeks of total treatment. He was well at 12 month follow-up.

#### Patient 4

A 79-year-old woman presented with *E. faecalis*-associated infective endocarditis affecting an implantable cardioverter defibrillator lead, which was complicated by discitis. She had a history of myocardial infarction, congestive heart failure, renal disease, hypertension and cancer. The defibrillator was extracted, and she was given ceftriaxone and amoxicillin as an inpatient. To facilitate early discharge, she received 1.5 g dalbavancin IV and oral amoxicillin. Eight weeks of antibiotics were used cumulatively. She achieved cure without adverse reactions and was well at 12 month follow-up.

#### Patient 5

A 31-year-old male IVDU with severe liver disease presented with MSSA-associated tricuspid valve endocarditis, complicated by cavitating lung lesions. He was treated conservatively using flucloxacillin and rifampicin over 4 weeks. As he insisted on early discharge, he was given two doses of 1.5 g dalbavancin IV 1 week apart, which led to cure without adverse reactions. He was uncontactable at 12 month follow-up but known to be alive.

#### Patient 6

A 64-year-old man with a history of aortic valve replacement, congestive cardiac failure, peripheral artery disease and renal disease presented with MSSA-associated aortic composite graft endocarditis. He was managed conservatively. Inpatient antibiotics included flucloxacillin and rifampicin, and he was discharged upon receiving two doses of 1.5 g dalbavancin IV 1 week apart and oral cefaclor long term, which led to cure without adverse reactions. He was well at 12 month follow-up.

#### Patient 7

A 73-year-old man with no previous cardiac surgery or comorbidities presented with uncomplicated *Streptococcus oralis*-associated aortic valve endocarditis. He underwent aortic valve replacement, was treated with amoxicillin as an inpatient and was discharged on dalbavancin and linezolid. The total duration of antibiotics was 6 weeks. He was cured without adverse reactions and well at 12 month follow-up.

#### Patient 8

An 80-year-old man with a history of aortic valve replacement, myocardial infarction, hypertension and stroke presented with *E. faecalis*-associated prosthetic valve endocarditis. He underwent redo aortic valve replacement and repair of an aorto-atrio fistula that developed as a complication of the endocarditis. He was treated with amoxicillin and ceftriaxone and discharged on two doses of 1.5 g dalbavancin 1 week apart and oral linezolid. The total duration of antibiotics was 6 weeks. He was cured without adverse reactions and was well at 12 month follow-up.

#### Patient 9

A 72-year-old man with a history of aortic valve replacement, myocardial infarction, renal disease, congestive cardiac failure, hypertension and diabetes presented with *S. oralis*-associated replacement aortic valve infective endocarditis. He received vancomycin and gentamicin, then amoxicillin and gentamicin as an inpatient. He was discharged with two doses of 1.5 g dalbavancin 1 week apart and oral amoxicillin to complete total 6 weeks of treatment. This led to cure without adverse reactions. He was well at 12 month follow-up.

#### Patient 10

An 81-year-old man with a history of renal disease, depression and cancer presented with *E. faecalis*-associated aortic valve endocarditis. He underwent valve replacement and was treated with amoxicillin as an inpatient and discharged on dalbavancin plus oral amoxicillin. The total duration of antibiotics was 6 weeks. He was cured without adverse reactions and was well at 12 month follow-up.

#### Patient 11

A 79-year-old man with a history of mild liver disease, hypertension and cancer presented with *E. faecalis*-associated aortic valve endocarditis. He received amoxicillin and ceftriaxone as an inpatient. Once stable, he was discharged with two doses of 1.5 g dalbavancin 1 week apart and oral amoxicillin to complete a total of 6 weeks of treatment. This led to improvement of symptoms without adverse reactions. He died 10 months after surgery of unrelated cancer.

### Conclusions

Infective endocarditis, a serious, complex disease associated with high mortality and morbidity,^1–4^ requires aggressive, often prolonged, specialized antibiotics and sometimes surgery. Conventional antibiotics with short half-lives often extend hospital stay. To enable outpatient completion of treatment, multidisciplinary teams can consider oral antibiotics and/or IV agents with longer half-lives, using OPAT clinics as required.

Dalbavancin’s long half-life of 14.4 days enables IV treatment as a single dose or two doses given 7–14 days apart.^5–7^ It can facilitate early discharge, freeing beds for patients in greater need and minimizing hospital stays. Although dalbavancin is not approved in infective endocarditis due to the absence of clinical trials, preclinical findings in animals and real-world evidence are encouraging,^11–16^^,^^20^ and expert opinion references dalbavancin in this setting.^18^^,^^19^

In our small cohort, dalbavancin was offered to personalize treatment to facilitate completion of treatment after discharge. Dalbavancin proved effective in all patients with no adverse reactions or early recurrence of infection.

Our study adds to the data supporting dalbavancin as a treatment for patients not suitable for conventional IV antibiotic regimens for infective endocarditis or in whom early discharge would be beneficial.
